# Supplementary material for: Extracting rubber tree parameters and estimating carbon storage using airborne LiDAR
Source: PLoS One. 2025 Aug 22;20(8):e0330768. doi: 10.1371/journal.pone.0330768 (PMC12373164; doi:10.1371/journal.pone.0330768)
Supplement: Attachment — Submitted filename: Response to Reviewers.docx [file pone.0330768.s003.docx]

Response to Reviewers

Dear Reviewers,

We deeply appreciate the effort and time that you have spent in reviewing our manuscript “Extracting Rubber Tree Parameters and Estimating Carbon Storage Using Airborne LiDAR” (ID: PONE-D-24-57180). We have studied the comments carefully and have made revisions which we hope meet with your approval. The revised outline is as follows:

We will add some quantitative results to the abstract section after the experiment. (Please refer to the abstract section)

We have added a discussion on the application of laser technology in the introduction section. (Please refer to the introduction section)

We have added some discussions on single tree segmentation methods based on deep learning methods. (Please refer to the section on single tree segmentation methods)

We have redesigned the method for estimating DBH. (Please refer to the DBH estimation method section in the methodology.)

We have made detailed revisions and expansions to the abstract, introduction, methodology, discussion, and conclusion.

We carefully checked the experimental results of the entire manuscript.

We carefully checked and modified the image format of the entire manuscript.

We have revised the entire manuscript and carefully addressed some terminology issues.

Response to reviewer 1 comments

Point 1: The title is inappropriate as it lacks an academic summary of the work. A mere introduction of the methodology fails to effectively encapsulate the thesis's theme.

Response 1: Thank you for helpful comment. We have revised the title of the article and adopted the comments of the reviewers.

Point 2: The introduction lacks a clear statement of the research objectives and further clarification is needed regarding the editorial contributions of this work.

Response 2: Thank you for helpful comment. We have made detailed revisions to the introduction. Please refer to the revised manuscript's introduction section, pages 2 to 5.

Point 3: The authors seem to have overlooked the comparison of their method with others, particularly in the discussion section, where a comprehensive discussion on the differences in accuracy, applicability, and other aspects of this work relative to other methods is missing.

Response 3: Thank you for helpful comment. We have added a deep learning based single tree segmentation method to our approach and conducted an analysis on it. For the method of estimating breast diameter parameters, we redesigned the experiment and constructed regression models such as power function, exponential function, linear function, and quadratic function by analyzing the parameters of east-west crown diameter, north-south crown diameter, and average crown diameter. These models were validated using indicators such as maximum, minimum, average error, and RMSE. In the discussion section, we have made modifications and expanded the methods. Please refer to section 3.4.3 of the paper, pages 13 to 14. Section 4.3, pages 18 to 21.

Point 4: Presenting part of the content in Section 4.3 in a tabular form would be more effective.

Response 4: Thank you for helpful comment. We have made detailed modifications to section 4.3 and presented it in the form of a table. Please refer to section 4.3, pages 18 to 21.

Point 5: The conclusion section is incomplete. While the authors mention an accuracy analysis, they fail to report the results of this analysis.

Response 5: Thank you for helpful comment. We have made revisions to the conclusion section. Please refer to the conclusion section, pages 26 to 27.

Point 6: The authors state in the conclusion that their method "provides a reliable solution," which lacks factual support. At least one corresponding validation of accuracy or applicability is required. If the authors wish to prove the reliability of their results, they need to conduct repeated experiments in at least three different applicable regions, as a single experiment cannot account for the impact of randomness.

Response 6: Thank you for helpful comment. We have made modifications to the method and discussed it. We have made revisions to the conclusion section. Please refer to the conclusion section, pages 26 to 27.

Response to reviewer 2 comments

Point 1: Clarify Novelty: The paper presents incremental improvements rather than a fundamentally new approach. The novelty claim needs to be reinforced by comparing the proposed DBH estimation method with other regression-based or machine-learning models. Otherwise, the study risks being viewed as a case study rather than a significant scientific breakthrough.

Response 1: Thank you for helpful comment. For the method of estimating breast height diameter parameters, we redesigned the experiment and constructed regression models such as power function, exponential function, linear function, and quadratic function by analyzing the parameters of east-west crown diameter, north-south crown diameter, and average crown diameter. These models were validated using metrics such as maximum, minimum, average error, and RMSE. In the discussion section, we made modifications and expanded the methods. Please refer to Section 4.3, pages 18-21 of the paper.

Point 2: Strengthen the Literature Review: The paper discusses previous work but does not sufficiently highlight how this study differs from existing methods. What specific gaps does this study fill? If similar segmentation methods have been applied in other forestry contexts, why is this study different?

Response 2: Thank you for helpful comment. We have made revisions to the introduction, methods, and discussion sections. We have strengthened the literature review and discussed our methods.

Point 3: Improve Readability & Academic Tone: The paper should undergo careful revision to simplify overly complex sentences, remove redundant explanations, and improve clarity. This will enhance accessibility without compromising technical depth.

Response 3: Thank you for helpful comment. We carefully revised the entire manuscript and modified some terminology. And simplified some complex sentences.

Point 4: Address Limitations More Explicitly: The discussion acknowledges the limitations of LiDAR, but the paper should go further by suggesting specific solutions. How can occlusion issues be mitigated? Would integrating multispectral or hyperspectral data improve results? How does tree density affect segmentation accuracy?

Response 4: Thank you for helpful comment. We have made modifications in the discussion section, discussing the limitations of lidar and providing our next research direction. Please refer to the discussion section.

Point 5: Provide a More Convincing Justification for Method Selection: The study evaluates three segmentation methods but does not clearly justify why these three were chosen over others. Were deep learning-based segmentation methods considered? If not, why?

Response 5: Thank you for helpful comment. We have added experiments based on deep learning to the single tree segmentation method and discussed its practicality. Please refer to section 3.4.3, pages 13 to 14, and the discussion section.

Response to reviewer 3 comments

Point 1: Please ensure that your manuscript meets PLOS ONE's style requirements, including those for file naming.

Response 1: Thank you for helpful comment. We have made modifications to the article according to the template, and the file name has also been changed.

Point 2: In your Methods section, please provide additional information regarding the permits you obtained for the work. Please ensure you have included the full name of the authority that approved the field site access and, if no permits were required, a brief statement explaining why.

Response 2: Thank you for helpful comment. We have added project sources in the methodology. Please refer to the overview section of the research area in the methodology, page 5.

Point 3: Please state what role the funders took in the study. If the funders had no role, please state: ""The funders had no role in study design, data collection and analysis, decision to publish, or preparation of the manuscript.

Response 3: Thank you for helpful comment. We have made modifications and added this explanation.

Point 4: We note that Figure 2 in your submission contain [map/satellite] images which may be copyrighted.

Response 4: Thank you for helpful comment. We have made modifications to Figure 2 and used our own orthorectified images as map data. The picture shows the orthophoto image captured by our own drone. (This image is not a map or satellite image, the copyright belongs to us.)

Point 5: Please upload a copy of Figure 9, to which you refer in your text on page 12. If the figure is no longer to be included as part of the submission please remove all reference to it within the text.

Response 5: Thank you for helpful comment. We have made modifications to Figure 9, please refer to the Methods section on page 11.
