# Supplementary material for: Extracting rubber tree parameters and estimating carbon storage using airborne LiDAR
Source: PLoS One. 2025 Aug 22;20(8):e0330768. doi: 10.1371/journal.pone.0330768 (PMC12373164; doi:10.1371/journal.pone.0330768)
Supplement: Attachment — Submitted filename: Response_to_Reviewers_auresp_2.docx [file pone.0330768.s004.docx]

Response to Reviewers

Dear Reviewers,

We deeply appreciate the effort and time that you have spent in reviewing our manuscript “Extracting Rubber Tree Parameters and Estimating Carbon Storage Using Airborne LiDAR” (ID: PONE-D-24-57180). We have studied the comments carefully and have made revisions which we hope meet with your approval. The revised outline is as follows:

We have made detailed revisions and expansions to the abstract, introduction, methodology, discussion, and conclusion.

We carefully checked the experimental results of the entire manuscript.

We carefully checked and modified the image format of the entire manuscript.

We have revised the entire manuscript and carefully addressed some terminology issues.

Response to reviewer 3 comments

Point 1: Abstract

1) Please make the introductory section of the abstract concise, it should be summarized in 3-4 lines

2) The abstracts section is a much longer, need to shorter

3) The abstract much cover the introduction and problem statement in 4-5 lines, the methodology (2-3 lines), major results/findings and discussion (4-6 lines) and importance/implication/application/conclusion (2-3 lines).

Response 1: Thank you for helpful comment. We have made revisions to the abstract, please refer to the abstract section for details, page 2.

Point 2: Introduction

1) Please include some numerical data with respect forest and climate change the following articles might be helpful.

3) Please include some numerical information on the status of forest carbon sequestration at global as well as China

4) Pleases enrich the introduction section with updated literature that why the use of technology and computing is needed in forest measurement.

5) Problem statement and justification section need modification supported by literature

Response 2: Thank you for helpful comment. We have made overall revisions to the introduction section. Added numerical information on forests and climate change, global carbon storage, and carbon neutrality. We explained from the perspective of forestry carbon sinks why technology and calculations are needed in forest measurement, and cited some references, pages 2 to 6.

Point 3: Discussion and conclusion

1) The discussion section need modification, the statement that belongs to author must be supported by the empirical data and those does not belong to author, a sources of citation might be provided. Also how the present findings are consistent or inconsistent with other must be provided in a logical, coherent and connected way.

2) Pleases include a limitation section in conclusion, also provide the importance of your study.

Response 3: Thank you for helpful comment. We have made modifications to the discussion and conclusion sections, pages 23 to 27.
